# The cognitive engine of evaluative threat: socially prescribed perfectionism and Music Performance Anxiety—a moderated mediation analysis of rumination and self-compassion

**DOI:** 10.3389/fpsyg.2026.1782906

**Published:** 2026-05-14

**Authors:** Haitao Yan, Chang Liu

**Affiliations:** 1 Hezhou University, Hezhou, Guangxi, China; 2 Chongqing University of Posts and Telecommunications, Chongqing, China

**Keywords:** cognitive interference, evaluative threat, moderated mediation, Music Performance Anxiety, rumination, self-compassion, Socially Prescribed Perfectionism

## Abstract

**Background:**

Socially Prescribed Perfectionism (SP) is a robust risk factor for Music Performance Anxiety (MPA), yet the underlying cognitive and regulatory mechanisms remain inadequately understood.

**Objective:**

This study aimed to delineate these pathways by constructing a moderated mediation model, examining rumination as a cognitive mediator and Self-Compassion (SC) as a multi-stage moderator.

**Methods:**

Data were collected from 606 university music students. Analyses were conducted using Hayes’ PROCESS macro (Model 59) to test the moderated mediation, controlling for academic grade and performance frequency.

**Results:**

Confirmatory factor analysis established an excellent measurement model fit (χ^2^/df = 4.37, CFI = 0.957, RMSEA = 0.075). SP significantly predicted MPA both directly and indirectly specifically through rumination; other ruminative dimensions (Symptom Rumination and Reflection) were non-significant mediators. Crucially, SC moderated this mediation process at multiple stages. First, SC buffered the initial impact of perfectionistic pressure on rumination (*B* = −0.10). Second, a complex “Awareness Paradox” emerged: the positive link between rumination and MPA was significantly stronger among individuals with high SC (*B* = 0.16). Consequently, conditional indirect effect analysis demonstrated that the indirect effect of SP on MPA via rumination was only significant at moderate and high levels of SC.

**Conclusion:**

This study validates the “social expectations” framework of MPA, identifying rumination as the critical mechanism translating internalized social pressure into cognitive interference. Theoretically, findings highlight the dual-edged nature of self-compassion: acting as a protective buffer against maladaptive thinking, while potentially facilitating heightened emotional awareness. Practically, music pedagogy should prioritize cognitive restructuring alongside nuanced self-compassion training to help musicians process, rather than suppress, performance-related distress.

## 1 Introduction

The pursuit of musical excellence is an arduous endeavor that frequently exposes performers to intense psychological scrutiny. Music Performance Anxiety (MPA) represents a significant and pervasive challenge within the performing arts community, affecting musicians across all levels of expertise (Kenny, 2011). This pervasive evaluative distress often manifests as MPA, a debilitating phenomenon characterized by a triad of physiological arousal, cognitive interference, and behavioral paralysis (Burin and Osório, 2017). In this context, negative emotions are conceptualized not merely as symptomatic outcomes of performance stress, but as fundamental cognitive-affective drivers that underpin the musician’s internal conceptualization of evaluative threat (Kenny, 2010). While nascent research has explored the symptomatic relief of MPA, a more profound inquiry is required to deconstruct the stable personality architectures and maladaptive cognitive processes that sustain this condition, particularly in elite training environments (Cox and Kenardy, 1993).

Central to the cognitive vulnerability of musicians is the construct of perfectionism. While the rigorous standards of classical music may foster high achievement, they also cultivate a “perfectionism paradox,” where the drive for excellence transitions into pathological concern (Dobos et al., 2019). Within the multidimensional framework of perfectionism, Socially Prescribed Perfectionism (SP)—the perceived mandate to meet the unrealistically high expectations of external authorities—emerges as the most clinically significant predictor of distress (Eysenck et al., 2007; Farley and Kelley, 2023). Unlike self-oriented strivings, SP is rooted in the fear of social alienation and perceived loss of agency, rendering the musician hyper-vigilant to signs of external disapproval (Frost et al., 1990).

However, the transition from a stable perfectionistic trait to acute performance anxiety is not direct; it is filtered through specific cognitive mechanisms. This study proposes that rumination, a maladaptive sub-type of rumination, serves as the primary “cognitive engine” of this process (Hewitt and Flett, 1991). Rumination involves a repetitive, gloomy preoccupation with perceived failures, which effectively drains the cognitive resources required for complex motor execution and artistic fluency (Kenny et al., 2004). Furthermore, the intensity of this pathological chain may be governed by individual differences in emotional regulation. Self-Compassion (SC), an integrated self-regulatory resource, offers a potential pivot point (Kenny et al., 2004). By shifting the internal dialogue from threat-defense to self-soothing, SC may redefine the boundary conditions of how perfectionistic pressure is processed (Osborne and Kenny, 2005).

To address these theoretical complexities, the current study constructs a moderated mediation model (Model 59). Our objective is to determine whether rumination mediates the impact of SP on MPA and to explore how Self-Compassion moderates the various stages of this psychological pathway

### 1.1 The externalized self: Socially Prescribed Perfectionism and MPA

In the context of classical music, where the “tyranny of the shoulds” often dominates student life, Socially Prescribed Perfectionism (SP) reflects the internalization of a critical audience (Neff, 2003). According to Self-Presentation Theory, musicians high in SP view every performance as a definitive test of their social worth. This externalization of standards creates a state of perpetual psychological precarity, where self-esteem is only as secure as the last performance (Neff and Vonk, 2009).

The contemporary educational climate—characterized by “Neijuan” (intense peer competition) and constant surveillance through social media recordings—further amplifies this vulnerability (Nolen-Hoeksema et al., 2008). Empirical evidence has consistently identified SP as a robust predictor of somatic and cognitive anxiety, as these individuals perceive the audience as a tribunal of critical judges rather than a supportive community (Osborne and Kenny, 2005). When the pressure to be perfect is perceived as a mandate from external authorities, the performer’s intrinsic artistic motivation is frequently replaced by a fear-based focus on error avoidance, directly escalating MPA.

*H1*: *Socially Prescribed Perfectionism (SP) will positively predict Music Performance Anxiety (MPA).*

### 1.2 Rumination as the mechanism of cognitive interference

To move beyond simple trait-anxiety correlations, we must examine the repetitive negative thinking that sustains distress. While some forms of reflection may be adaptive for professional growth, rumination—characterized by passive and gloomy self-comparison against unachievable ideals—serves as a primary mechanism of cognitive interference (Papageorgi et al., 2007).

Based on Attentional Control Theory, we posit that rumination consumes the limited cognitive resources (e.g., working memory and executive function) required for the complex motor and expressive demands of musical performance (Patston and Osborne, 2016). For the socially prescribed perfectionist, SP provides the impossible benchmark, while rumination keeps the perceived failure active in the consciousness, even in the absence of an immediate audience (Schlenker and Leary, 1982). This maladaptive cycle effectively “drains” the cognitive capacity needed for flow, resulting in the technical and emotional “choking” characteristic of MPA.

*H2*: SP will positively predict rumination.

*H3*: Rumination will mediate the relationship between SP and MPA.

### 1.3 Self-compassion as a regulatory pivot: the awareness paradox

The impact of perfectionism and rumination on anxiety may be contingent upon the individual’s self-regulatory framework. Self-Compassion (SC) involves treating one’s inadequacies with kindness, acknowledging common humanity, and maintaining mindful awareness (Shafran et al., 2002). According to Gilbert’s Three-Circle Model, SC activates the mammalian “soothing system, which can downregulate the “threat system” triggered by perfectionistic failure (Stoeber and Otto, 2006).

However, the role of SC in performance settings may be more complex than simple buffering. While self-kindness reduces the frequency of self-criticism, the mindfulness component of SC encourages an open, non-judgmental awareness of emotional states (Treynor et al., 2003). This leads to what we term the “Awareness Paradox”: self-compassionate individuals may be more accurately attuned to their emotional responses, acknowledging the link between rumination and anxiety rather than masking it through avoidant strategies (Watkins, 2004). To explore these sophisticated boundary conditions, we test whether SC moderates the paths from SP to rumination and from rumination to MPA.

*H4*: Self-Compassion (SC) will moderate the mediation process at multiple stages (Model 59), redefining the individual’s psychological relationship with perfectionistic pressure and ruminative thoughts.

**FIGURE 1:**
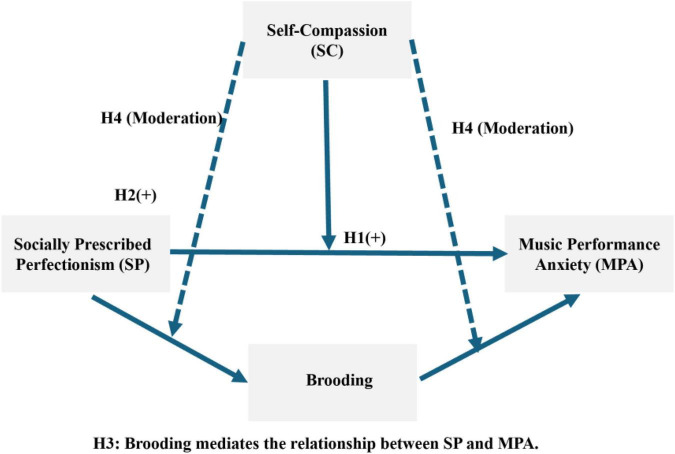
The hypothesized moderated mediation model. SP, Socially Prescribed Perfectionism; MPA, Music Performance Anxiety; SC, Self-Compassion. H1, H2, and H3 represent the paths for the mediation model; H4 (dashed lines) represents the hypothesized moderating effects of Self-Compassion on all constituent paths of the mediation (as per Hayes’ Model 59). Standardized coefficients and control variables are omitted for visual clarity.

Figure 1 illustrates the hypothesized moderated mediation model of the present study, constructed according to Hayes’ (2018) Model 59 framework. In this conceptual model, Socially Prescribed Perfectionism (SP) serves as the independent variable, while Music Performance Anxiety (MPA) is the primary outcome. The mediation component of the model comprises three hypothesized paths: the direct effect of SP on MPA (H1), the impact of SP on the cognitive mediator, rumination (H2), and the subsequent effect of rumination on MPA (H3).

To explore the boundary conditions of this mechanism, Self-Compassion (SC) is integrated as a multi-stage moderator (H4). Specifically, the model tests whether SC moderates all constituent pathways of the mediation process, including the direct path from SP to MPA, the pro-mediational path (SP→ Rumination), and the post-mediational path (Rumination → MPA). For visual clarity, demographic control variables (e.g., grade and performance frequency) are omitted from the diagram but were included in all subsequent statistical analyses.

## 2 Materials and methods

### 2.1 Participants

In this study, participants were recruited from university music departments using a targeted sampling approach. To ensure high engagement and data quality, the questionnaires were administered in controlled classroom settings under the supervision of departmental faculty and researchers. Initially, 612 university students completed the survey. After screening the collected data for duplicate responses, logical inconsistencies, or non-consent, 6 samples were excluded. This rigorous screening process resulted in 606 valid responses, achieving a 99.02% validity rate. The participant pool consisted of 178 males (29.4%) and 428 females (70.6%), with a mean age of 19.20 ± 1.01 years; specifically, 35.5% were below 19 years old, and 64.5% were 20 years or older. Academic year distribution included 215 first-year (35.5%), 126 second-year (20.8%), 192 third-year (31.7%), and 73 fourth-year (12.0%) students. Further demographic details are presented in Table 1.

**TABLE 1:** Demographic characteristics of participants.

Variable	Category	Frequency (n)	Percentage (%)
Gender	Male	178	29.4%
	Female	428	70.6%
Age	Below 19	215	35.5%
	20 and above	391	64.5%
Academic year	First-year	215	35.5%
	Second-year	126	20.8%
	Third-year	192	31.7%
	Fourth-year	73	12.0%

### 2.2 Instruments

The following self-report instruments were utilized in this study:

#### 2.2.1 Social perfectionism

Participants’ levels of socially prescribed perfectionism were assessed using the Social Perfectionism subscale of the Multidimensional Perfectionism Scale (Zhang et al., 2013; Hewitt and Flett, 1991; Zhang et al., 2013). The original scale comprises 33 items across three dimensions: self-oriented, socially prescribed, and other-oriented perfectionism. This study exclusively employed the 9-item socially prescribed perfectionism subscale, with responses rated on a 7-point Likert scale ranging from 1 (strongly disagree) to 7 (strongly agree). In the present study, the Social Perfectionism subscale demonstrated good internal consistency, with a Cronbach’s alpha coefficient of 0.841.

#### 2.2.2 Kenny Music Performance Anxiety Inventory (K-MPAI, 2009 Version) (MPA)

Music performance anxiety was measured using the Kenny Music Performance Anxiety Inventory (K-MPAI, 2009 version) (Kenny, 2023), developed by Kenny (2009) and translated into Chinese by Sun (2020) (Pan et al., 2025), which has demonstrated robust psychometric properties. This 40-item scale utilizes a 7-point Likert scale, from 0 (not at all true) to 6 (very true). It encompasses three main dimensions: Family Background (FB), General Psychological Vulnerability (GV), and Performance Anxiety (PA), which further branch into 12 sub-dimensions. These include intergenerational transmission of anxiety and parental empathy within FB; controllability, depression, hopelessness, and trust within GV; and somatic anxiety, pre- and post-performance reflection, self/other scrutiny, performance outcome concerns, memory reliability, and commitment to performance within PA. The K-MPAI exhibited excellent internal consistency in this study, with a Cronbach’s alpha coefficient of 0.940.

#### 2.2.3 Ruminative Response Scale (RRS)

Ruminative thinking was evaluated using a Chinese revision of Nolen-Hoeksema’s Ruminative Response Scale (Han and Yang, 2009) (Han and Yang, 2009). This version comprises 22 items across three dimensions: Symptom Rumination (12 items), Rumination, and Reflection. Responses were recorded on a 4-point Likert scale, ranging from 1 (never) to 4 (always), with higher total scores indicating more severe ruminative thinking. The scale’s three dimensions are Symptoms, Rumination, and Reflection. In the current study, the RRS showed excellent internal consistency, with a Cronbach’s alpha coefficient of 0.969.

#### 2.2.4 Self-Compassion Scale—Short Form (SCS-SF)

Self-compassion was assessed using the Self-Compassion Scale—Short Form (SCS-SF). This 12-item scale measures six dimensions: self-kindness, self-judgment, common humanity, isolation, mindfulness, and over-identification. Items are rated on a 5-point Likert scale, from 1 (almost never) to 5 (almost always). Items belonging to self-judgment, isolation, and over-identification (6 items in total) are reverse-scored. To obtain a total self-compassion score, the average scores of all six dimensions are calculated. Higher scores indicate greater self-compassion. The SCS-SF items are derived from the long form, and its translation into Chinese for this study referenced the version by Chen et al. (2011) and Huang et al. (2023). Table 2 shown composition of the questionnaire.

**TABLE 2:** Composition of the questionnaire.

Instrument	Dimensions	Number of items	Cronbach’s alpha (α)
Social Perfectionism Scale (SP)	Socially prescribed perfectionism	9	0.841
Kenny Music Performance Anxiety Inventory (K-MPAI)	Family background (FB), general psychological vulnerability (GV), performance anxiety (PA)	40	0.940
Ruminative Response Scale (RRS)	Symptom rumination, rumination, reflection	22	0.969
Self-Compassion Scale—Short Form (SCS-SF)	Self-kindness, self-judgment, common humanity, isolation, mindfulness, over-identification	12	0.882

### 2.3 Data collection and procedure

Data were collected between November 1 and November 30, 2025, employing a targeted sampling strategy within university music departments. To ensure high data quality and a controlled response environment, questionnaires were administered during specialized music classes with the support of departmental faculty. Prior to completing the survey, all participants provided informed consent, confirming their understanding of voluntary participation and the option to withdraw at any time.

Stringent data screening was applied, leading to the exclusion of responses with unusually short completion times, straight-line patterns, or internal logical inconsistencies. This process yielded 606 valid questionnaires out of 612 initially collected, resulting in a 99.02% validity rate. This high rate is attributed to the supervised, classroom-based administration method.

### 2.4 Statistical analysis strategy

Descriptive statistics were computed using SPSS 26.0 to characterize participant demographics and the distribution of all primary variables. Structural Equation Modeling (SEM) was conducted using AMOS 26.0, encompassing both measurement and structural model analyses. Maximum Likelihood (ML) estimation was employed for model fit assessment.

To maintain an optimal ratio of observed variables to sample size and to enhance model parsimony, we employed the item parceling technique for multi-dimensional scales (e.g., K-MPAI and RRS). Specifically, the sub-scale scores were used as indicators for their respective latent constructs. For the unidimensional Social Perfectionism scale, items were randomly parceled into three indicators. In the structural model, each latent construct was assigned a unique set of indicators with distinct labels to ensure local independence and avoid artificial correlations. No single item or indicator was reused across different latent variables, ensuring the integrity of the construct validity.

### 2.5 Ethics statements

All procedures performed in studies involving human participants were with the ethical standards of the institutional and/or national research committee and with the 1964 Helsinki Declaration and its later amendments or comparable ethical standards. This study was approved by the Ethics Committee of the Hezhou University (IRB No. 20250530).

### 2.6 Reliability and validity analysis

**FIGURE 2:**
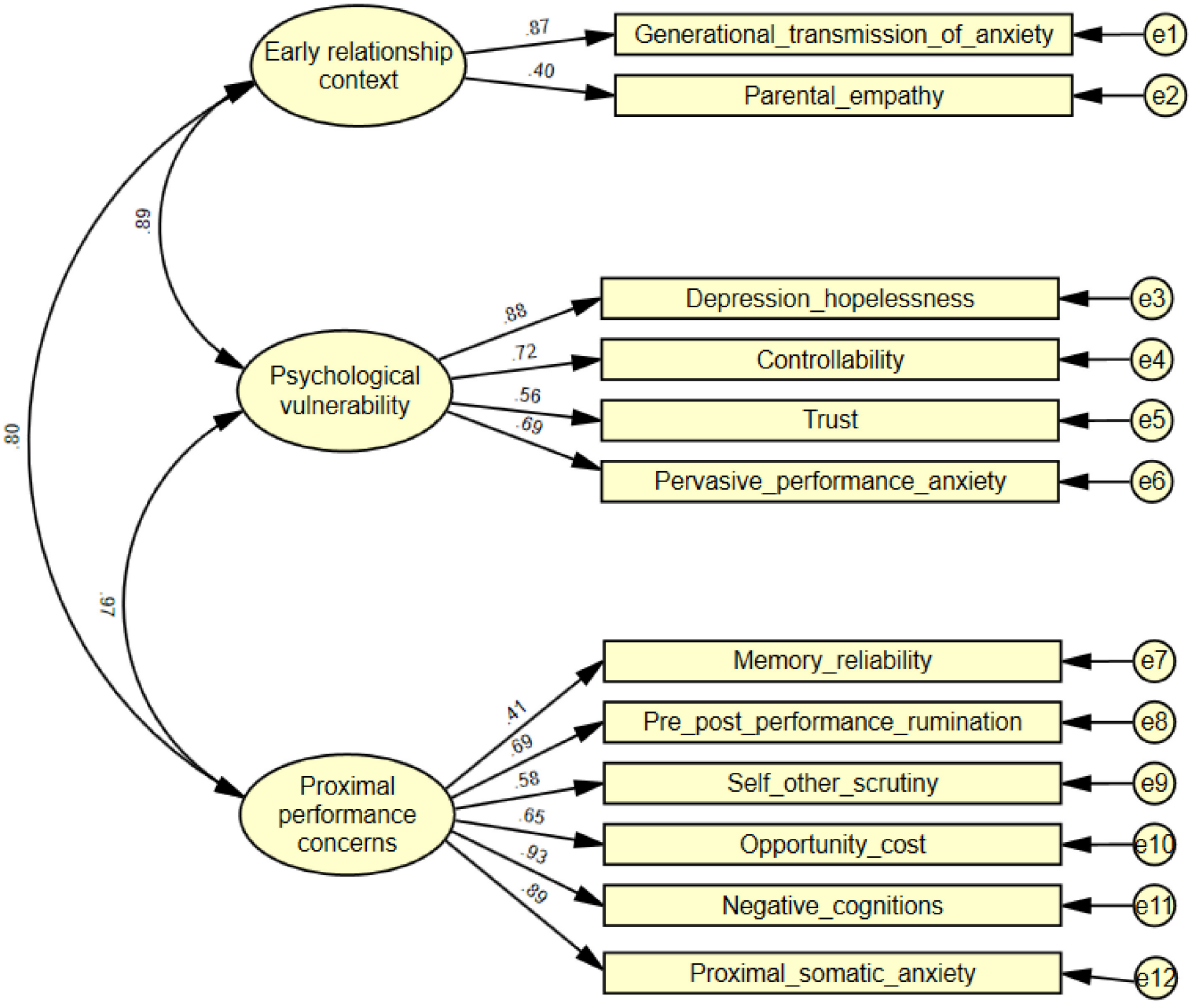
Confirmatory Factor Analysis (CFA) measurement model.

Prior to testing the substantive hypotheses, Confirmatory Factor Analysis (CFA) was performed to establish the psychometric properties of the measurement model. The CFA results, depicted in Figure 2, indicated a good fit to the data, with all key indices meeting acceptable standards. Specifically, the chi-square to degrees of freedom ratio (χ^2^/df = 4.371) was within the commonly accepted range (typically below 5), the Comparative Fit Index (CFI = 0.957) and Goodness-of-Fit Index (GFI = 0.939) both exceeded the 0.90 criterion, and the Root Mean Square Error of Approximation (RMSEA = 0.075) and Standardized Root Mean Square Residual (RMR = 0.065) were well below the 0.08 guideline. These values collectively attest to the measurement model’s robust fit to the observed data.

## 3 Results

### 3.1 Common method bias (CMB) test

Since all data in this study were collected through self-report measures, potential common method bias (CMB) was addressed using both procedural and statistical controls. Procedurally, we ensured participant anonymity, emphasized that there were no “right” or “wrong” answers, and incorporated reverse-coded items within the scales to reduce response sets and social desirability bias.

Statistically, to provide a more rigorous and sensitive assessment beyond the limitations of Harman’s single-factor test, we employed the Unmeasured Latent Method Factor (ULMF) approach within the Confirmatory Factor Analysis (CFA) framework (Podsakoff et al., 2003). We compared the fit indices of our hypothesized measurement model with two competing models: (1) a single-factor model where all items loaded onto one general latent factor, and (2) a method-factor model that included an additional latent method factor loading on all observed indicators.

As shown in Table 3, the single-factor model yielded a poor fit (χ^2^/*df* = 24.257,*CFI* = 0.676,*RMSEA* = 0.196), which was substantially worse than the hypothesized model (χ^2^/*df* = 4.371,*CFI* = 0.957,*RMSEA* = 0.075). Furthermore, the results of the ULMF test indicated that adding the latent method factor did not lead to a significant improvement in model fit (Ä*CFI* = 0.004,Ä*RMSEA* = 0.002,Ä*SRMR* = 0.003), with all changes in fit indices remaining well below the 0.01 threshold. These findings collectively suggest that common method variance does not exert a substantial influence on the relationships between our variables, confirming the integrity of our data.

**TABLE 3:** Comparison of the hypothesized and single-factor models (CMB Test).

Model	χ^2^/d*f*	CFI	GFI	AGFI	NFI	RMSEA
Hypothesized model	4.098	0.949	0.918	0.878	0.937	0.080
Single-factor model	24.257	0.676	0.642	0.506	0.180	0.196
Method-factor model (ULMF)	4.362	0.961	0.942	0.880	0.062	0.073

### 3.2 Descriptive statistics and preliminary analysis of group differences

The means and standard deviations for all primary variables are summarized in Table 5. Preliminary analyses, including independent samples *t*-tests and one-way ANOVAs, were conducted to examine potential differences across demographic groups (Gender, Grade, and Performance Frequency).

As shown in Table 4, no significant gender differences were observed across any of the study variables (*p* > 0.05). However, significant variations across academic years (Grade) were found for almost all constructs, including general psychological vulnerability (GV), performance anxiety (PA), socially prescribed perfectionism (SP), self-compassion (SC), and all three dimensions of ruminative thinking (*p* < 0.05). Additionally, performance frequency significantly influenced levels of general psychological vulnerability (*F* = 3.301, *p* < 0.05) and performance anxiety (*F* = 5.474, *p* < 0.01).

**TABLE 4:** Descriptive statistics and group differences of study variables.

Variable	Mean	S.D.	Gender		Grade		Frequency	
			*F/t*	*p*	*F/t*	*p*	*F/t*	*p*
Family background (FB)	3.23	1.08	0.079	0.937	0.939	0.421	1.582	0.192
General vulnerability (GV)	3.49	0.99	0.025	0.980	5.017	0.002	3.301	0.020
Performance anxiety (PA)	3.76	1.00	1.290	0.198	5.372	0.001	5.474	0.001
Social perfectionism (SP)	3.55	0.92	1.188	0.235	2.764	0.041	1.170	0.320
Symptom rumination	2.09	0.73	0.459	0.647	4.153	0.006	1.157	0.326
Reflection	2.14	0.77	1.725	0.085	3.864	0.009	1.378	0.248
Rumination	2.18	0.82	0.326	0.745	3.835	0.010	2.421	0.065
Self-compassion (SC)	3.27	0.55	1.165	0.245	3.801	0.010	1.086	0.354

Bold values indicate significant differences (*p* < 0.05).

**TABLE 5:** Correlation matrix and discriminant validity (fornell-larcker criterion).

Variable	1	2	3	4	5	6	7	8
1. Family background (FB)	1	1	1	1	1	1	1	1
2. General vulnerability (GV)	0.492***							
3. Performance anxiety (PA)	0.343***	0.745***						
4. Social perfectionism (SP)	0.207***	0.596***	0.601***					
5. Symptom rumination	0.384***	0.688***	0.574***	0.544***				
6. Reflection	0.336***	0.620***	0.507***	0.516***	0.879***			
7. Rumination	0.338***	0.657***	0.586***	0.553***	0.906***	0.884***		
8. Self-compassion (SC)	−0.410***	−0.553***	−0.474***	−0.363***	−0.519***	−0.401***	−0.473***	

***p < 0.001.

To ensure the internal validity of the structural model and to account for potential confounding effects, Grade and Performance Frequency were included as covariates in all subsequent structural equation modeling (SEM) analyses.

### 3.3 Bivariate correlation analysis

Pearson correlation analysis was conducted using SPSS 27.0 to examine the interrelationships among the study variables. The results, summarized in Table 5, revealed that all primary constructs were significantly correlated in the hypothesized directions (*p* < 0.001).

Specifically, the three dimensions of music performance anxiety (Family Background, General Vulnerability, and Performance Anxiety) demonstrated moderate to strong positive correlations with socially prescribed perfectionism and the three dimensions of ruminative thinking (Symptom Rumination, Reflection, and Rumination). Notably, General Vulnerability and Performance Anxiety were highly correlated (*r* = 0.745,*p* < 0.001), underscoring their close psychological link. In contrast, Self-Compassion was significantly and negatively associated with all other variables, including anxiety dimensions, perfectionism, and rumination, suggesting its potential role as a protective factor. These robust bivariate relationships provide a solid statistical foundation for the subsequent testing of the structural model.

### 3.4 Mediation analysis

To further elucidate the psychological mechanism through which socially prescribed perfectionism influences music performance anxiety (MPA), we performed a parallel mediation analysis using the PROCESS macro for SPSS (Model 4; Hayes, 2018). Based on the theoretical framework and preliminary correlation findings, we controlled for gender, age, grade, and performance frequency. Additionally, the Family Background and General Vulnerability dimensions of the K-MPAI were included as covariates to isolate the unique predictive variance of Social Perfectionism on the behavioral and cognitive aspects of performance anxiety.

The mediation results, based on 5,000 bootstrap samples, indicated that the three dimensions of ruminative thinking functioned differently within the model. Specifically, neither Symptom Rumination [Effect = 0.014, 95% CI (−0.004, 0.034)] nor Reflection [Effect = 0.005, 95% CI (−0.013, 0.026)] reached statistical significance as mediators, as their respective confidence intervals included zero.

**FIGURE 3:**
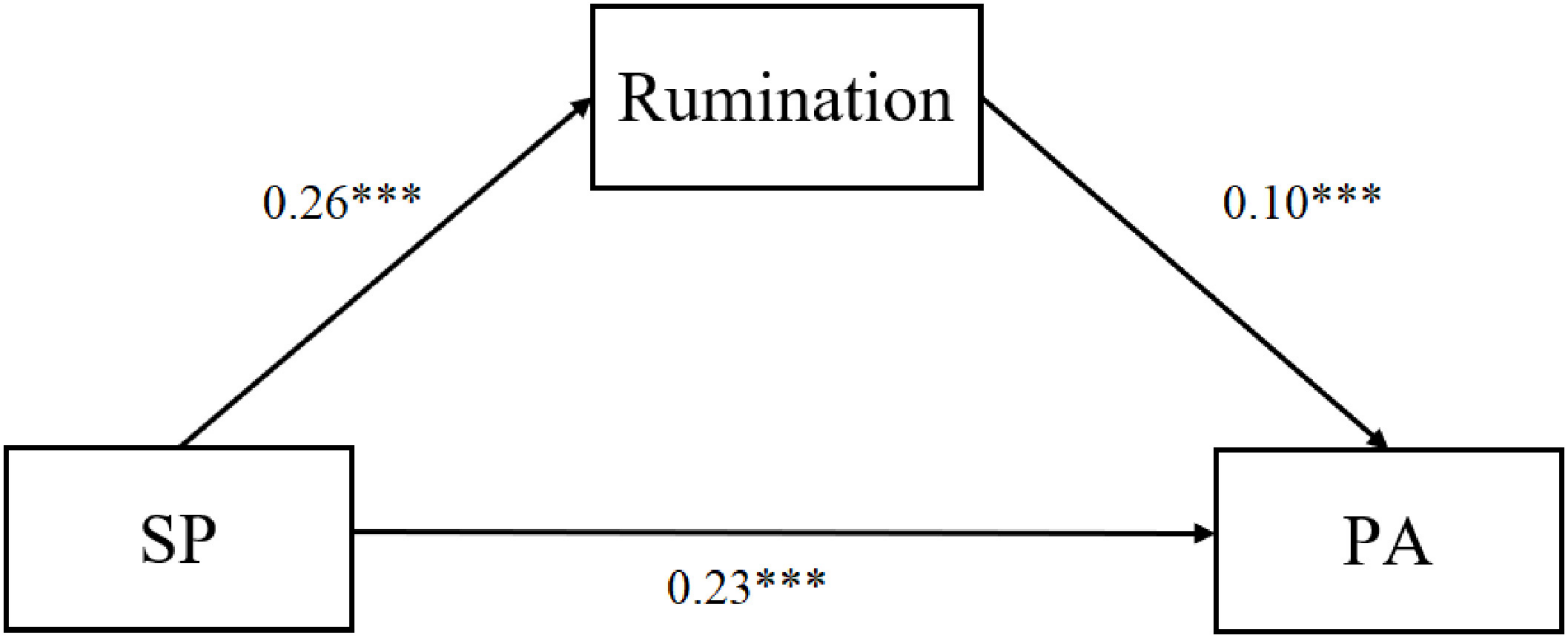
The mediating role of rumination in the relationship between socially prescribed perfectionism and music performance anxiety. ***p < 0.001.

In contrast, Rumination emerged as a significant mediator in the relationship between Social Perfectionism and MPA. As illustrated in Figure 3, Social Perfectionism significantly and positively predicted both rumination (β = 0.26,*p* < 0.001) and MPA (β = 0.23,*p* < 0.001). Furthermore, rumination was found to be a significant positive predictor of MPA (β = 0.10,*p* < 0.006).

The total, direct, and indirect effects are detailed in Table 6. The results confirmed a significant indirect effect of Social Perfectionism on MPA through rumination [Effect = 0.03, Boot SE = 0.01, 95% CI (0.004, 0.05)]. Since the direct effect of Social Perfectionism remained significant (β = 0.25,*p* < 0.001), these findings suggest that rumination partially mediates the impact of perfectionistic social expectations on music performance anxiety. Specifically, socially prescribed perfectionism not only directly heightens anxiety levels but also exacerbates performance anxiety indirectly by increasing the individual’s tendency toward maladaptive rumination.

**TABLE 6:** Mediation effect of rumination between social perfectionism and MPA.

Effect	Effect	se	*t*	*p*	LLCI	ULCI
Total effect	0.27	0.04	7.84	< 0.001	0.21	0.34
Direct effect	0.25	0.04	6.84	< 0.001	0.18	0.32
Effect	Effect	BootSE	BootLLCI		BootULCI	
Indirect effect	0.03	0.01	0.004		0.05	

LLCI and ULCI refer to the lower and upper limits of the 95% confidence interval, respectively.

### 3.5 Testing for the moderated mediation model

**FIGURE 4:**
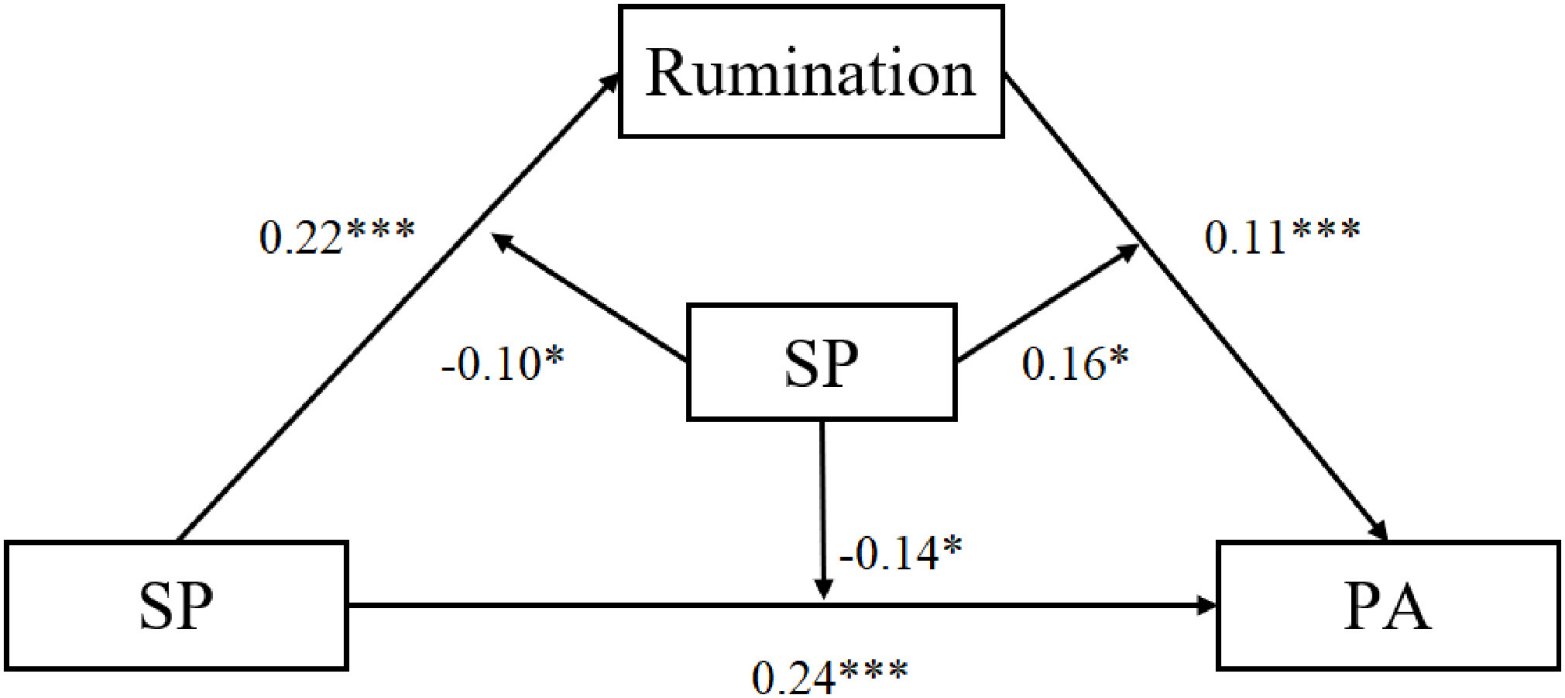
Structural model of the moderated mediation analysis. *p < 0.05, ***p < 0.001.

To further explore the boundary conditions of the mediating mechanism, we employed Hayes’ (2018) PROCESS macro (Model 59) to test a moderated mediation model. This model examined whether Self-Compassion (SC) moderated: (a) the relationship between Social Perfectionism (SP) and Rumination (Path *a*); (b) the direct effect of SP on Performance Anxiety (PA) (Path *c’*); and (c) the relationship between Rumination and PA (Path *b*). All continuous predictors were standardized prior to analysis to facilitate interpretation and reduce multicollinearity. The results are summarized in Table 7 and illustrated in Figure 4.

**TABLE 7:** Results of the moderated mediation model (Model 59).

Predictive variable	Rumination			Performance anxiety (PA)		
	B	SE	t	B	SE	t
Social Perfectionism (SP)	0.22	0.03	6.68***	0.24	0.04	6.78***
Rumination				0.11	0.04	2.64**
Self-compassion (SC)	−0.23	0.05	−4.35***	−0.14	0.06	−2.47*
SP × SC	−0.10	0.04	−2.37*	−0.13	0.06	−2.24*
Rumination × SC				0.16	0.06	2.58*
R^2^		0.50			0.62	
F		65.34***			89.30***	

*N* = 606. Standardized coefficients are reported. *p < 0.05, **p < 0.01, ***p < 0.001.

#### 3.5.1 Moderation of the pro-mediational and direct paths

As shown in Table 7, the regression model predicting rumination was significant (*R*^2^ = 0.50,*F* = 65.34,*p* < 0.001). Social Perfectionism positively predicted rumination (*B* = 0.22,*p* < 0.001), while Self-Compassion served as a significant negative predictor (*B* = −0.23,*p* < 0.001). Crucially, the interaction between SP and SC was significant (*B* = −0.10,*p* < 0.02), indicating that Self-Compassion buffered the impact of Social Perfectionism on Rumination. Simple slope analysis revealed that as Self-Compassion increased, the predictive power of Social Perfectionism on Rumination weakened (Conditional effects: β_*Low*_ = 0.27,β_*Mean*_ = 0.22,β_*High*_ = 0.16; all *p* < 0.05).

In the second model predicting Performance Anxiety (*R*^2^ = 0.62,*F* = 89.30,*p* < 0.001), Social Perfectionism (*B* = 0.24,*p* < 0.001) and Rumination (*B* = 0.11,*p* < 0.009) both acted as positive predictors. The interaction between SP and SC significantly predicted PA (*B* = −0.13,*p* < 0.025), suggesting that the direct effect of perfectionistic expectations on anxiety is mitigated by higher levels of self-compassion. Furthermore, the interaction between Rumination and SC was also significant (*B* = 0.16,*p* < 0.010).

#### 3.5.2 Analysis of conditional indirect effects

The conditional indirect effect analysis (Table 8) further clarified how Self-Compassion fluctuates the mediation process. At low levels of Self-Compassion, the indirect effect of Social Perfectionism on Performance Anxiety via rumination was not statistically significant [*Effect* = 0.006,95%*CI*(−0.026,0.0390)]. However, the indirect effect became significant at mean [*Effect* = 0.025,95%*CI*(0.001,0.051)] and high levels of Self-Compassion [*Effect* = 0.034,95%*CI*(0.006,0.066)]. These findings suggest a “moderated mediation” effect, wherein the psychological pathway from perfectionism through rumination to performance anxiety is significantly altered by an individual’s level of self-compassion.

**TABLE 8:** Conditional indirect effects of SP on PA via rumination.

Level of Self-compassion	Effect	Boot SE	Boot LLCI	Boot ULCI
Low (−1 SD)	0.0066	0.016	−0.0267	0.0396
Mean	0.0253	0.012	0.0009	0.0509
High (+1 SD)	0.0340	0.015	0.0057	0.0658

## 4 Discussion

The primary objective of this study was to elucidate the internal mechanisms linking Socially Prescribed Perfectionism (SP) to Music Performance Anxiety (MPA) among university music students. Specifically, we examined the mediating role of rumination and the multidimensional moderating role of Self-Compassion (SC). The findings provide empirical support for a moderated mediation model (Model 59), offering a nuanced understanding of how maladaptive cognitive styles and self-regulatory resources interact to influence performance-related distress.

### 4.1 The effect of socially prescribed perfectionism on MPA

Consistent with our hypotheses and the multidimensional perfectionism model (Hewitt and Flett, 1991; Mor et al., 1995), the results demonstrated that Socially Prescribed Perfectionism is a significant positive predictor of MPA (Path ć). This finding aligns with the notion that SP—characterized by the belief that others (e.g., mentors, peers, audiences) hold unrealistically high standards for oneself—is a particularly toxic cognitive vulnerability factor (Kenny et al., 2004). Unlike self-oriented perfectionism, which can sometimes drive artistic excellence, SP involves a perceived lack of autonomy and a chronic sense of potential social evaluation (Nolen-Hoeksema et al., 2008). In the context of music education, musicians with high SP likely perceive the stage as a site of potential “execution” of their social reputation rather than a supportive artistic community, thereby triggering the sympathetic arousal associated with MPA (Stoeber and Eismann, 2007; McNeil et al., 2022).

### 4.2 The mediating role of rumination

A central finding of this study is that rumination significantly mediates the relationship between SP and MPA, whereas Symptom Rumination and Reflection did not reach statistical significance. This provides a precise answer to the “how” of the perfectionism-anxiety link. Musicians high in SP tend to internalize external pressures, leading to a specific maladaptive ruminative style—rumination—which involves passive and gloomy self-comparison against unachievable standards (Short and Mazmanian, 2013).

Our path analysis confirms that SP significantly elevates rumination, which in turn acts as a proximal catalyst for performance anxiety. While reflection may involve adaptive problem-solving, rumination traps the musician in a “why” cycle that consumes cognitive resources (e.g., working memory) required for musical execution (Treynor et al., 2003). This creates a debilitating cycle where the perceived external pressure fuels internal cognitive interference, ultimately manifesting as acute anxiety.

### 4.3 The dual-stage moderating role of Self-Compassion

The study extended previous research by exploring how Self-Compassion (SC) functions as a moderator across multiple paths of the mediation model. First, the results showed that SC significantly buffers the impact of SP on Rumination (*B* = −0.10,*p* < 0.05). By treating oneself with kindness rather than judgment, musicians can mitigate the development of obsessive rumination even when under high external pressure (Nolen-Hoeksema et al., 2008).

However, the moderation analysis revealed a complex interaction in the second stage of the model (*Brooding*→*PA*). Contrary to the typical “buffering” hypothesis, our simple slope analysis indicated that the positive relationship between Rumination and MPA was *stronger* at high levels of self-compassion (*B* = 0.16, *p* < 0.05). While this appears counter-intuitive, it may reflect the specific nature of emotional awareness in self-compassionate individuals (Eysenck et al., 2007). Those low in SC often employ avoidance or suppression strategies (Joormann et al., 2011), which may temporarily blunt the statistical association between their conscious reporting of “rumination” and “anxiety” (Neff, 2003). Conversely, individuals with high SC practice mindfulness and non-judgmental awareness; they are more accurately attuned to their emotional states, acknowledging the anxiety that follows maladaptive thinking rather than avoiding it (Neff et al., 2007). This suggests that for self-compassionate musicians, the pathway from thought to emotion is more coherent and acknowledged (Raes, 2010).

### 4.4 Theoretical and cultural implications: “Neijuan” and the digital panopticon

Theoretically, this study integrates the Social Expectations Model of perfectionism with Self-Compassion Theory in the context of the performing arts. In the current Chinese educational environment, the phenomenon of “Neijuan” (involution) and the “Digital Panopticon”—where every performance is potentially recorded and critiqued online—amplifies the pressure of Social Perfectionism (Huawei and Jenatabadi, 2024).

The validation of the moderated mediation model suggests that while high-stakes performance environments are pervasive, individual self-regulatory resources like self-compassion can fundamentally change how these pressures are processed. Crucially, the identification of conditional indirect effects shows that the mediation through rumination is only significant at moderate and high levels of SC. This suggests that self-compassion does not simply “cancel” anxiety; rather, it transforms the individual’s relationship with their internal cognitive states, facilitating a move from avoidant processing to integrated emotional awareness (Ma et al., 2026).

### 4.5 Practical implications and limitations

Practically, these results suggest that interventions for musicians should not only focus on technical mastery but also on cognitive restructuring to reduce SP (MacBeth and Gumley, 2012). Moreover, while cultivating self-compassion is beneficial for reducing the overall frequency of rumination (Gross and John, 2003), educators should be aware that self-compassionate students may be more sensitive to their emotional fluctuations. They may need support in accepting and “moving through” these acknowledged emotions rather than being taught to suppress them (Shafran et al., 2002; Grecucci et al., 2015).

Limitations include the cross-sectional design, which precludes causal inference (Maxwell and Cole, 2007). Despite this, our CMB tests (section 3.1) suggest that common method bias was not a critical issue (Podsakoff et al., 2003). Future research should employ longitudinal designs or incorporate physiological measures (e.g., heart rate variability) to complement self-reports (Thurber et al., 2010), and further explore how these entrenched mechanisms differ between music students and professional performers (Spahn et al., 2010).

### 4.6 Future research directions

Despite the theoretical and practical contributions of this study, several limitations offer compelling avenues for future investigation. First, the cross-sectional design precludes definitive causal inferences regarding the “Perfectionism → Rumination → Anxiety” pathway. While our moderated mediation model is theoretically grounded, future research should employ longitudinal designs or cross-lagged panel analysis to track the developmental trajectory of these variables across an entire concert season or academic year.

Second, while our Common Method Bias (CMB) tests confirmed data integrity, the reliance on self-report measures remains a limitation. Future studies should integrate physiological markers of stress—such as Heart Rate Variability (HRV), skin conductance, or salivary cortisol levels—to complement subjective reports of performance anxiety. This multi-method approach would be particularly valuable in further exploring the “Awareness Paradox” identified in our moderation analysis (对应3.5), helping to determine whether self-compassionate musicians experience lower biological arousal even when reporting higher conscious emotional awareness.

Third, the sample was limited to university music students. Professional orchestral musicians face distinct pressures, such as career tenure and high-stakes financial consequences, which may entrench their coping mechanisms differently. Comparative studies between student and professional populations would enhance the generalizability of the model.

Finally, future research should move toward intervention-based designs. Having established the buffering role of self-compassion, researchers could implement Mindful Self-Compassion (MSC) training programs or randomized controlled trials (RCTs) to test whether cultivating self-compassion directly reduces the frequency of rumination and mitigates the debilitating effects of perfectionism in real-time performance settings. Such evidence would provide the “gold standard” for clinical applications in music pedagogy.

## 5 Conclusion

The present study delineates a sophisticated psychological mechanism linking Socially Prescribed Perfectionism (SP) to Music Performance Anxiety (MPA) among university music students. By validating a moderated mediation model, we have identified that rumination serves as the critical “cognitive engine” through which external perfectionistic pressures are transformed into acute performance anxiety. Specifically, the internalization of unrealistic social standards fuels a maladaptive ruminative style, which in turn consumes the cognitive resources essential for high-stakes musical execution.

Furthermore, this research underscores the nuanced, dual-stage regulatory role of Self-Compassion (SC). While self-compassion functions as a robust “psychological shield” that buffers the initial impact of perfectionism on rumination, it also fundamentally alters the individual’s relationship with their internal emotional states. The discovery of the “Awareness Paradox”—where higher self-compassion strengthens the statistical link between rumination and felt anxiety—suggests that self-compassionate musicians move away from avoidant coping toward a more integrated, mindful awareness of their emotions.

In the competitive climate of modern music education (e.g., “Neijuan”), these findings suggest that fostering psychological resilience is as vital as technical mastery. Interventions should prioritize reducing the internalization of perfectionistic standards and cultivating self-compassion to break the cycle of maladaptive rumination. Ultimately, transitioning from a “perfection-oriented” to a “compassion-oriented” mindset may allow musicians to process their performance-related fears more coherently, facilitating long-term artistic growth and psychological wellbeing.

## Data Availability

The original contributions presented in this study are included in the article/supplementary material, further inquiries can be directed to the corresponding author.
